# Dynamic and reversible transcriptomic age shifts induced by COVID-19 in Korean whole blood

**DOI:** 10.18632/aging.206270

**Published:** 2025-06-10

**Authors:** Kyungwhan An, Yoonsung Kwon, Jihun Bhak, Hyojung Ryu, Sungwon Jeon, Dougu Nam, Jong Bhak

**Affiliations:** 1 Korean Genomics Center (KOGIC), Ulsan National Institute of Science and Technology (UNIST), Ulsan 44919, Republic of Korea; 2 Department of Biomedical Engineering, College of Information-Bio Convergence Engineering, Ulsan National Institute of Science and Technology (UNIST), Ulsan 44919, Republic of Korea; 3 Clinomics Inc., Ulsan 44919, Republic of Korea; 4 Geromics Inc., Suwon 16229, Republic of Korea; 5 AgingLab, Ulsan 44919, Republic of Korea; 6 Department of Biological Sciences, College of Information-Bio Convergence Engineering, Ulsan National Institute of Science and Technology (UNIST), Ulsan 44919, Republic of Korea

**Keywords:** aging clock, whole blood, RNA-seq, COVID-19, anti-aging

## Abstract

We developed the first genome-wide transcriptomic clock specific to Korean ethnicity to predict chronological age using whole blood samples from 440 healthy individuals. Our analysis revealed profound age acceleration – up to 21.31 years – during homeostatic disruption in COVID-19 patients, which reverted to baseline upon recovery. These findings highlight the ability of the blood transcriptome to dynamically track reversible changes in age-associated inflammatory responses during infections. Our study underscores the potential of anti-aging interventions in managing infectious diseases.

## INTRODUCTION

The aging clock is a machine learning model that estimates biological age based on omics data, capturing molecular changes beyond chronological age [1]. DNA methylation has been widely used as a primary aging biomarker [2, 3]. However, its gene regulatory effects remain poorly understood, complicating biological interpretation [4, 5]. Gene expression offers a more functionally relevant biomarker with enhanced temporal resolution compared to CpG site-based measures [6–8]. This enables gene expression to reflect whole-body health conditions in real physiological time with greater acuity.

Certain genes exhibit consistent expression changes in blood throughout the human lifespan, correlating with age-related phenotypes such as IL-6 levels and muscle strength [9–11]. Building on these findings, Peters et al. (2015) pioneered a blood transcriptomic clock based on 1,497 genes associated with chronological age from large-scale microarray data. The clock showed stronger correlations with age-related blood traits, such as blood pressure and cholesterol, than with chronological age [12]. Ren and Kuan (2020) introduced a concept of transcriptomic age acceleration analogous to epigenetic age acceleration. Here, they illustrated accelerated age among cancer subtypes measured by multi-tissue RNA clocks [13]. Moreover, Holzscheck and colleagues (2021) inferred accelerated transcriptomic age in skin fibroblasts from patients with Hutchinson–Gilford Progeria Syndrome (HGPS) and reduced transcriptomic aging in mice undergoing caloric restriction (CR) [14]. Recent advancements have expanded transcriptomic clocks to single-cell resolution to scrutinize the cell-type-specific progression of biological aging [15, 16].

Despite these developments, the application of RNA clocks to systematically investigate transcriptomic age shifts caused by disease states remains limited, particularly in East Asian populations. Robust biomarkers associated with these shifts also remain underexplored.

This study fills these critical gaps by leveraging bulk mRNA sequencing to investigate the transcriptomic age shifts in blood during both transient (COVID-19) and chronic (Mental illnesses) pathological states. We also identify novel aging biomarkers to provide deeper molecular insight into the aging process.

## RESULTS

### Whole blood mRNA accurately predicts chronological age in healthy individuals

**Figure 1:**
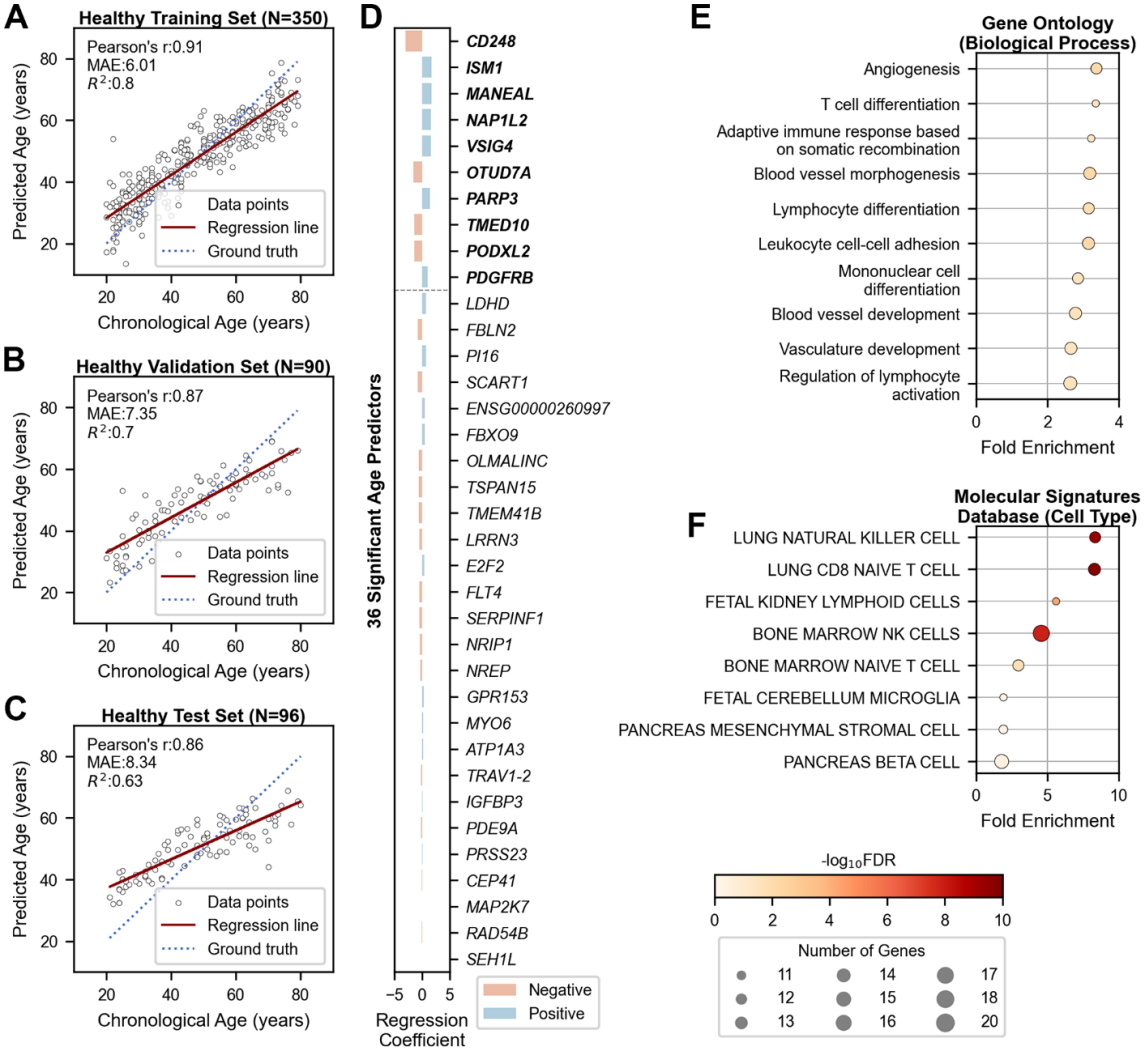
**Chronological age prediction using 36 genes in healthy cohorts.** (**A**–**C**) Scatter plots showing the performance of the age prediction model on (**A**) training (N=350), (**B**) validation (N=90), and (**C**) independent test (N=96) data. The x-axis shows chronological age, and the y-axis shows predicted age based on the mRNA clock. Each sample is represented by an open black dot, with a solid red line indicating the regression trend and a dotted blue line indicating perfect correlation. (**D**) Bar plot showing genes ranked by their importance in age prediction. The x-axis shows regression coefficients, and the y-axis lists the gene symbols of the 36 age-predictive genes. The top ten genes are shown in bold. Blue and red bars indicate positive and negative associations with aging, respectively. (**E**, **F**) Dot plots displaying gene-set enrichment results of the 36 age-predictive genes with their 180 co-expressed genes based on (**E**) Gene Ontology (Biological Processes) and (**F**) Molecular Signatures Database (Cell Type). The x-axis represents fold enrichment, and the y-axis portrays the top ten annotated biological functions, sorted by fold enrichment (FDR < 0.05). Dot color denotes the statistical significance, and dot size indicates the number of enriched genes. MAE = Mean Absolute Error; r = Pearson’s Correlation Coefficient; R^2^ = Coefficient of Determination; FDR = False Discovery Rate.

We developed a machine learning model to predict transcriptomic age using bulk RNA sequencing data from 350 healthy individuals of Korean ethnicity (Supplementary Figure 1). From 13,834 stably expressed genes in whole blood, 301 genes were significantly correlated with chronological age (|r| > 0.35 and FDR < 0.05, Supplementary Table 1). Using the LARS LASSO method, we constructed a linear prediction model that selected 36 genes with strong predictive power. The model achieved high accuracy in the training cohort (*R^2^*_train_ = 0.80; Figure 1A) and robust performance in validation and test cohorts of 90 and 96 individuals, respectively (*R^2^*_validation_ = 0.70 and *R^2^*_test_ = 0.63; Figure 1B, 1C). Consistent age-gene correlations were observed across all cohorts (Supplementary Figure 2).

Our clock outperformed existing transcriptomic aging clocks for Korean samples but performed less effectively on Caucasian samples compared to Ren Clock trained on the same ethnicity (GSE134080; Supplementary Figure 3). These results emphasize the necessity of population-specific models, justifying the development of a tailored RNA clock for accurate age prediction in Korean individuals.

The 36 age-predictive genes, ranked by regression coefficients, revealed both positive and negative associations with aging (Figure 1D and Supplementary Table 2). Gene-set enrichment analysis with 180 co-expressed genes highlighted angiogenesis and lymphoid immunity as the dominant pathways (FDR < 0.05; Figure 1E, 1F). A two-dimensional t-SNE plot of the 36 genes revealed moderate stratification according to age groups, but did not differentiate between sex groups within the embedded space (Supplementary Figure 4).

### Transcriptomic age acceleration in response to COVID-19 and mental illnesses

**Figure 2:**
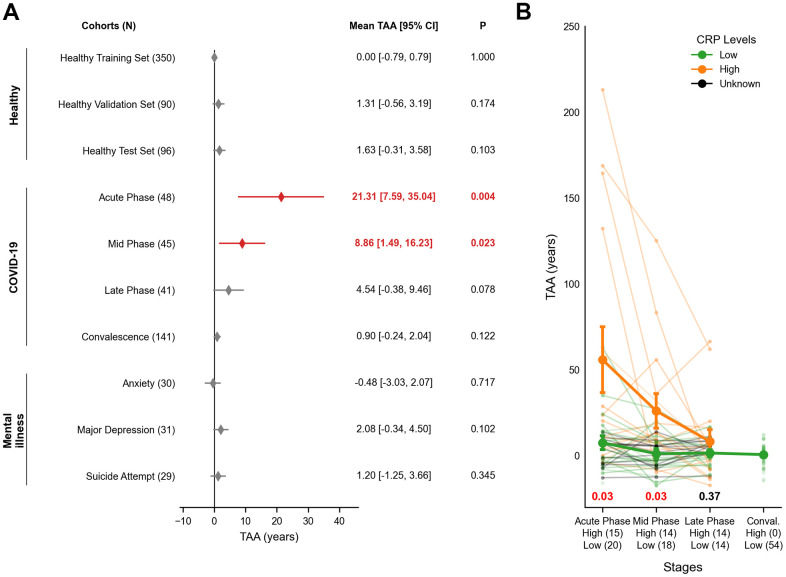
**Transcriptomic age acceleration (TAA) across healthy, COVID-19 and mental illness cohorts.** (**A**) A forest plot illustrates interval estimates of TAA across cohorts. Red-filled diamonds indicate statistically significant TAA (*P* < 0.05), while grey-filled diamonds denote no significance. The x-axis represents TAA in years, and the y-axis lists the study cohorts. Nominal P-values for TAA are displayed on the right-hand side, with bold red figures indicating statistical significance. (**B**) A line plot depicts TAA trajectories in COVID-19 patients, stratified by CRP levels (Low, High, and Unknown). The x-axis shows infection stages with respective sample sizes of High and Low CRP groups, while the y-axis displays TAA in years. Bold lines represent the group trends for High CRP (orange) and Low CRP (green) groups. Error bars indicate the mean ± SEM. Nominal P-values are shown at the bottom for each phase using two-sided Welch's t-test. Red indicates statistical significance while black shows no significance. The group trend of Unknown CRP (green) was omitted. SEM = Standard Error of Sample Means; TAA = Transcriptomic Age Acceleration.

The 36-gene clock was applied to disease cohorts for analysis. Despite a near-uniform age distribution across cohorts (Supplementary Figure 5), the clock failed to accurately predict the chronological age of certain unhealthy individuals (Supplementary Figure 6). We quantified transcriptomic age acceleration (TAA) to measure the deviation between chronological and transcriptomic age. Healthy cohorts showed no significant age acceleration (Figure 2A; Healthy). Mean TAA of the healthy cohorts in validation was no more than 0.98 years (95% CI: -0.79 to 3.6, FDR = 0.199), supporting their non-diseased status (Supplementary Table 3).

In SARS-CoV-2 infection, longitudinal samples showed significant TAA during the acute phase (mean TAA = 21.31 years; 95% CI: 7.59 to 35.04, *P* = 0.004). This dramatically declined in mid (8.86 years; 95% CI: 1.49 to 16.23, *P* = 0.023) and late phases (4.54 years; 95% CI: -0.38 to 9.46, *P =* 0.078). Notably, an independent cohort of 141 convalescent samples showed no evidence of acceleration (0.90 years; 95% CI: -0.24 to 2.04, *P* = 0.122) (Figure 2A; COVID-19). Consistent with these findings, TAA was negatively correlated with the time since infection, indicating a gradual return to transcriptomic homeostasis (Regression coefficient = -8.49, *P* = 0.02; Supplementary Figure 7). Expression dynamics of the 36 blood aging biomarkers in COVID-19 mirrored these trends, with VSIG4 levels declining during acute phases and recovering over time, independent of stage-specific differentially expressed genes (Supplementary Figure 8 and Supplementary Table 4).

COVID-19 patients with higher inflammatory status, indicated by C-reactive Protein (CRP), showed significantly elevated TAA during acute and mid phases (*P =* 0.03 for both), while no differences were observed in the late phase (*P* = 0.37; Figure 2B). TAA was also associated with higher neutrophil counts, lower lymphocyte counts, and declining serum albumin levels (*P* < 0.05; Supplementary Figure 9).

In psychiatric cohorts, TAA was modest and statistically insignificant overall (mean TAA = 0.94 years; 95% CI: -3.03 to 4.50, FDR = 0.199; Supplementary Table 3). These results suggest that acute infection drives transient TAA more strongly than chronic conditions such as mental illnesses.

### Validation of transcriptomic age acceleration in public infection cohorts

To validate the dynamics of transcriptomic age acceleration (TAA), we analyzed publicly available RNA-seq datasets from two independent cohorts: COVID-19-related acute respiratory distress syndrome (ARDS; GSE273149) and Hepatitis C Virus infection (HCV; GSE119117).

**Figure 3:**
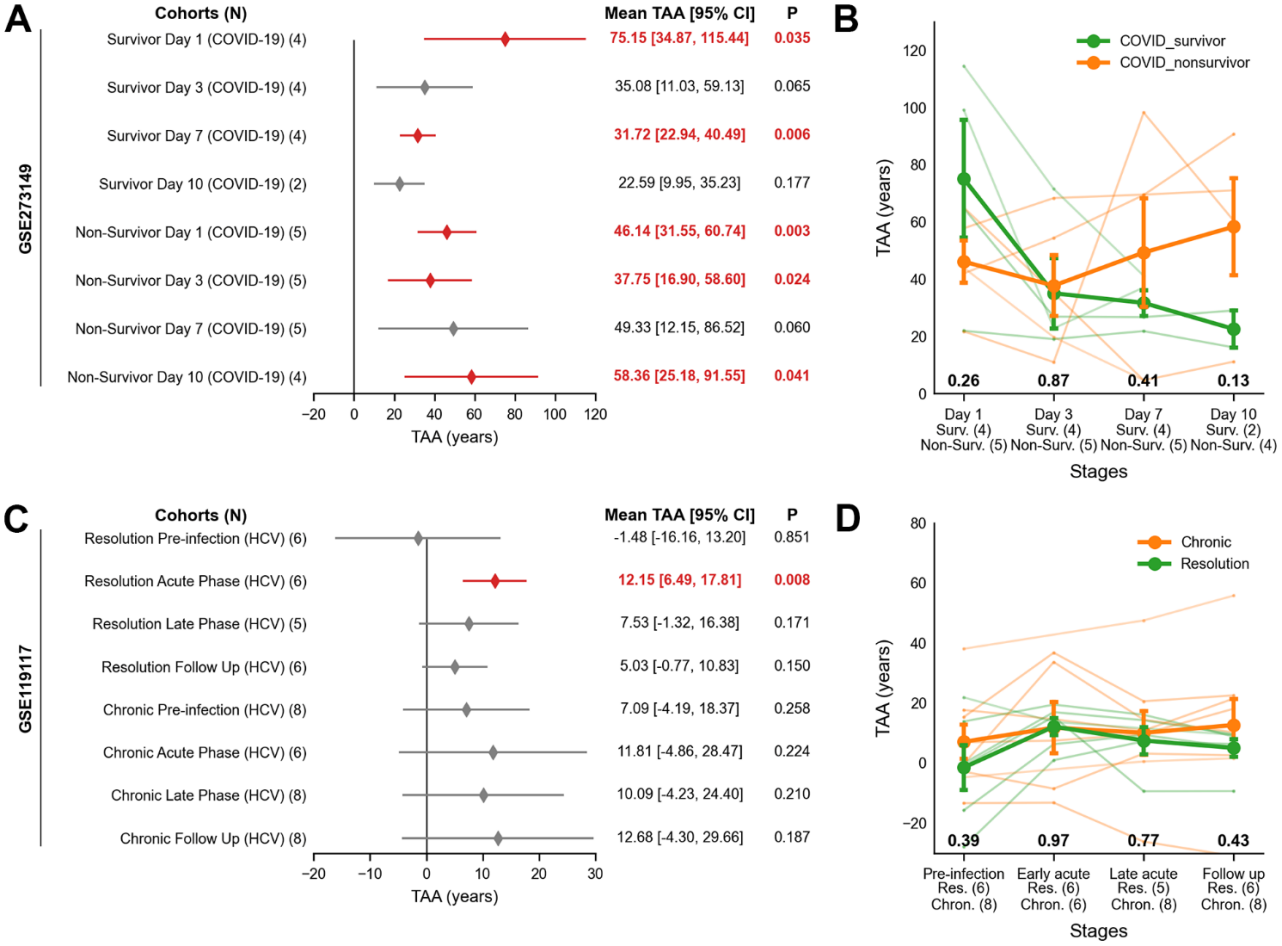
**Transcriptomic age acceleration (TAA) across COVID-19 ARDS and HCV cohorts using publicly available RNA-seq data.** (**A**, **B**) COVID-19 ARDS cohort. (**A**) Forest plot showing the interval estimates of TAA. Statistically significant TAA values (*P* < 0.05) are indicated by red-filled diamonds, while non-significant values are marked with grey-filled diamonds. The x-axis represents TAA in years, and the y-axis shows cohort labels. Statistical results for TAA are listed on the right, with significant values in bold red. (**B**) Line plot of TAA trajectories stratified by clinical outcome (survivors, orange; non-survivors, green). The x-axis indicates infection stages with sample sizes; the y-axis shows TAA in years. Lines represent group means ± SEM; nominal *P*-values from two-sided Welch’s t-test are shown below each phase. (**C**, **D**) HCV cohort. (**C**) Forest plot showing TAA in years with significance as in (**A**). (**D**) Line plot of TAA trajectories stratified by resolution stage (chronic, orange; resolution, green), with interpretation as in (**B**). ARDS = Acute Respiratory Distress Syndrome, Chron. = Chronic; HCV = Hepatitis C Virus; Non-Surv. = Non-Survivor; Res. = Resolution; SEM = Standard Error of Sample Means; Surv. = Survivor; TAA = Transcriptomic Age Acceleration.

In the COVID-19 ARDS cohort, non-survivors exhibited persistent TAA across all measured time points. On Day 1, TAA was 46.14 years (95% CI: 31.55 to 60.74, *P* = 0.003), remaining elevated on Day 3 (37.75 years; 95% CI: 16.90 to 58.60, *P* = 0.024) and Day 7 (49.33 years; 95% CI: 12.15 to 86.52, *P* = 0.060). By Day 10, TAA increased further (58.36 years; 95% CI: 25.18 to 91.55, *P* = 0.041), reflecting unresolved systemic inflammation and failure to recover (Figure 3A). Survivors, in contrast, showed high TAA on Day 1 (75.15 years; 95% CI: 34.87 to 115.44, *P* = 0.035) but showed a progressive return to baseline by Day 7 (31.72 years; 95% CI: 22.94 to 40.49, *P* = 0.006) and Day 10 (22.59 years; 95% CI: 9.95 to 35.23, *P* = 0.177), indicating recovery (Figure 3A). Although not statistically significant, COVID-19 survivors exhibited an initial increase in TAA compared to non-survivors on Day 1 (+13.86 years, *P* = 0.26), which reversed upon Day 10 (-35.77 years, *P* = 0.13; Figure 3B). Accordingly, TAA trajectories in survivors trended downward to baseline, whereas non-survivors showed sustained elevation at Day 7 (*P*_interaction_ = 0.047; Supplementary Table 5).

In the HCV cohort, TAA was 12.15 years (95% CI: 6.49 to 17.81, *P* = 0.008) in the acute phase of the resolved cases, decreasing to 5.03 years (95% CI: -0.77 to 10.83, *P* = 0.150) during follow-up. Pre-infection samples showed negligible TAA (-1.48 years, 95% CI: -16.16 to 13.20, *P* = 0.47). Chronic HCV cases exhibited stable TAA across all stages, with no significant changes (*P* > 0.05, Figure 3C). Across all stages, there was no evidence of TAA difference between resolution (green) and chronic (orange) groups (*P* > 0.05, Figure 3D). TAA trajectories in HCV showed no significant distinction between the subgroups (*P*_interaction_ > 0.05, Supplementary Table 5).

## DISCUSSION

Our study provides compelling evidence for the reversibility of transcriptomic age in response to systemic stressors such as infections [17, 18]. In whole blood, transcriptomic age exhibits dynamic and transient shifts that are independent of chronological age. These shifts likely reflect deviations from a healthy state rather than permanent changes in biological age, given that the clock was exclusively trained on the chronological age of non-diseased individuals. Notably, transcriptomic age closely tracked the inflammatory course of COVID-19 patients from acute illness through recovery. Among clinical markers, C-reactive Protein (CRP) exhibited the strongest correlation with transcriptomic age acceleration (TAA). CRP is a well-established inflammation marker linked to increased all-cause mortality, including sepsis-related deaths [19, 20].

Early inflammatory response is critical in determining later disease outcomes [21]. In COVID-19, a robust initial inflammatory surge facilitates viral clearance and recovery, while a delayed or insufficient response can lead to prolonged systemic inflammation and adverse clinical outcomes [22]. Despite the small sample size, the COVID-19 ARDS cohort demonstrated that survivors exhibited a sharp inflammatory surge which resolved to baseline, indicating effective immune activation. Non-survivors had a blunted response with persistently high TAA, reflecting immune failure and disease progression. Taken together, blood transcriptomic age is a useful proxy for age-associated inflammatory responses, offering insights into disease progression through the lens of aging biology.

CXCL9, a plasma protein previously implicated in inflammation and experimentally validated to promote endothelial cell senescence [23], showed a positive correlation with chronological age in our whole blood data. However, the strength of this correlation was insufficient for inclusion in our analysis (Supplementary Table 6). Instead, *VSIG4* emerged as a key surrogate marker of blood aging. VSIG4, a potent negative regulator of pro-inflammatory macrophages and T-cells, demonstrated significant downregulation during recovery, indicating a reduced inflammatory environment [24, 25]. Alongside *NREP*, a gene included in the 36-gene set, *VSIG4* has been identified as a deleterious signature of aging across multiple tissues and species [26].

Viruses act as pro-aging factors. Virus-induced senescence (VIS) is linked to disease severity through senescence-associated secretory phenotypes (SASPs), which drive systemic inflammation [27]. While causality remains to be established, our results suggest that infection-induced changes in aging biomarkers challenge the notion of aging as merely a risk factor for infection susceptibility [28]. Recent studies indicate that senolytics mitigate complications of viral infections, highlighting their therapeutic potential [29, 30]. Moreover, our findings support the role of anti-aging interventions in improving vaccine efficacy [31]. Taken together, these results underscore the potential for repurposing anti-aging interventions as complementary strategies to enhance resilience and health outcomes in the context of infectious diseases.

Mental health issues have been associated with epigenetic age acceleration [32], and chronic psychosocial stress has been implicated in epigenetic aging [33]. However, our results did not align with these findings. We suggest that the transcriptomic clock may be more sensitive to acute stress, such as COVID-19, rather than chronic stress, such as mental health disorders. Future research should involve larger sample sizes and classify patients based on clinically approved indices to better establish a definitive relationship.

Our study highlights significant challenges in predicting transcriptomic age across diverse ethnic groups. A clock trained on a single ethnic group failed to generalize across populations, with our Korean-trained clock overestimating age in Caucasian COVID-19 patients (Supplementary Figure 10) and performing poorly on the predominantly Caucasian GTEx dataset (Supplementary Figure 11). This mirrors Ren & Kuan’s observation that ethnicity-matched models minimize error [13], indicating that blood aging signatures are compounded by ethnic-specific genetic, environmental, and socioeconomic factors [34, 35]. Moreover, technical variability in RNA sequencing – such as differences in RNA quality, sample handling, and sequencing platforms – introduces batch effects that exacerbate prediction errors. In future, comprehensive batch correction methods, such as ComBat-seq and RUVSeq, should be systematically employed in both intra- and inter-ethnic contexts to ensure reproducible cross-cohort age prediction [36, 37].

Recent studies propose that aging clocks reflect stochastic molecular variation, or entropy, accumulated over time [38, 39]. While our clock is primarily driven by inflammation, inflammation itself may amplify transcriptional variability [40, 41], implying that stochasticity is a core component of the blood transcriptomic clock presented here. Although we have utilized LASSO regression to prioritize highly performant features of age prediction, we cannot confidently claim that all 36 genes, including *VSIG4* and *NREP*, reflect programmed aging. Future studies are warranted to elucidate the variance explained by entropic aging in the clock, particularly at the single-cell level to resolve cellular heterogeneity that is masked in bulk transcriptomic data as used in our study.

## MATERIALS AND METHODS

### Study population and sample collection

We collected a total of 559 whole blood samples from healthy donors who participated in the Korean Genome Project (KGP) with no apparent disease onset at the time of blood draw [42, 43]. Additionally, we obtained 124 whole blood samples from the Mental Health Cohort [44]. From the COVID-19 Infection and Recovery Cohorts, we collected 146 and 141 whole blood samples, respectively. Out of the 146 COVID-19 Infection Cohort samples, 134 samples were longitudinally collected from 48 subjects over a one-month period, covering the acute (N=48), mid (N=45), and late (N=41) phases of infection (unpublished).

### Bulk mRNA sequencing using illumina sequencers

Whole blood samples collected in PAXgene® Blood RNA Tubes were stored frozen at -80° C. Total RNA extraction utilized the PAXgene Blood RNA Kit from Qiagen following the manufacturer’s protocol. RNA quality was assessed by analyzing 1 μl on the Bioanalyzer system (Agilent) to ensure RNA Integrity Number (RIN) and rRNA ratio met required standards. We used 100 ng of total RNA for library preparation with the TruSeq RNA Library Prep Kit and TruSeq Stranded mRNA Sample Preparation Kit (Eukaryote) for the HiSeq2500 and NovaSeq5000 platforms, respectively, following the manufacturer’s instructions. Library quality was assessed with the Agilent 2100 BioAnalyzer and quantified using the KAPA library quantification kit (Kapa Biosystems). Paired-end (2×101 or 2×151) RNA sequencing was performed on HiSeq2500 and NovaSeq5000 sequencers.

### Bulk mRNA sequencing using BGI/MGI sequencers

To enrich polyadenylated mRNA and deplete rRNA, we used the Dynabeads mRNA Purification Kit (Invitrogen). Libraries were assessed for size distribution using the Agilent D1000 ScreenTape. Library preparation was conducted using BGI’s custom protocol or the MGIEasy RNA Directional RNA Library Prep Set (BGI) for the BGISeq500 and DNBSEQ-T7 platforms, respectively, following manufacturer protocols. Library quantification was performed using the Qubit 2.0 Fluorometer with the Qubit DNA HS Assay kit (Thermo Fisher Scientific). Paired-end (2×100 or 2×150) RNA sequencing was conducted on the DNBSEQ-T7RS (MGI) platform.

### Quality check and expression quantification

Sequenced RNA reads had adapters removed and were filtered for low-quality reads using fastp (version 0.23.1) with default options [45]. The filtered RNA reads were aligned to the human reference genome FASTA (GRCH38 p.13) using STAR (version 2.7.10b) with default settings [46]. Only those samples with Q30 Rate > 0.90, GC Rate > 0.46, and Total Mapping Rate > 70% were included (Supplementary Tables 7, 8). Transcripts and respective genes were annotated with their Ensembl ID and gene symbol using the annotation GFF3 file (GENCODE version 43) and RSEM (version 1.3.3) [47]. We removed any genes with duplicate gene symbols. Raw expression of each gene was estimated by RSEM (version 1.3.3) with default parameters [48]. DESeq2 (version 1.42.0; R package) was used to normalize the expression counts for sequencing depth and RNA library composition [49]. To normalize raw counts from publicly available RNA-seq data, size factors were computed using the geometric means of genes across samples in the Korean study population with the “geoMean” argument in the “estimateSizeFactors” function of the DESeq2 package.

### Expression count preprocessing

To ensure stable mRNA signals, genes with a median expression of zero were removed. Then, we removed the genes with median expression below 20. This reduced the number of input genes from 69,222 to 13,834. The remaining genes had their expression values standardized across the samples to Z-scores using “preprocessing.StandardScaler” (scikit-learn version 1.3.2). The mean and standard deviation for the scaler were calculated using the training dataset only.

### Sample selection for training the age prediction model

We randomly selected samples from our RNA-seq dataset to achieve a near-uniform age distribution. Of the initial 440 samples, 350 were assigned to the training dataset and 90 to the validation dataset in an 80:20 ratio using “model_selection.train_test_split” (scikit-learn version 1.3.2). The split was stratified into six age group bins using “np.digitize” (numpy version 1.26.2). From the principal component analysis (PCA) using all 13,834 genes, we separated out the cluster of 90 samples with distinct batch information and expression profiles – sequencing performed in 2019 by BGISeq500 platform. PCA was performed using “decomposition.PCA” from scikit-learn (version 1.3.2) for each cohort.

### Finding age-associated genes via simple correlation analysis

DESeq2-normalized expression values of each gene were correlated with chronological age using Pearson’s test, restricted to the 350 samples in the training dataset to prevent data leakage. P-values were adjusted for multiple tests using the Benjamini-Hochberg approach with “stats.multitest.fdrcorrection” (statsmodels version 0.14.0). Genes with |r| > 0.35 and FDR < 0.05 were considered significantly associated with chronological age.

### Korean blood transcriptomic clock

The LARS (Least Angle Regression) LASSO (Least Absolute Shrinkage and Selection Operator) model was trained on 350 healthy samples the genes of significant age correlation using “linear_model.LassoLarsIC” (scikit-learn version 1.3.2) with default parameters. Here, we assume that the combined effect of age-predictive genes on the sample age is simply a linear combination of their expression. Given our sample size, we proceeded the feature selection with information criterion (asymptomatically equal to Leave-one-out cross-validation) to prevent over- or under-fitting [50]. For detecting the optimal regularization strength (i.e., alpha), we chose a model with the lowest value of Bayesian information criterion (BIC) by iteratively minimizing the BIC (Supplementary Table 9).

### Peters and ren blood transcriptomic clock

Transcriptomic age by Peters Clock was calculated using “TranscriptomicPredictionModel” function from BioLearn [51]. Ren Clock was calculated using “RNAAgeCalc” function from racpy [13] with following options: tissue = “blood”, stype = “Caucasian”, and signature = “GTExAge”.

### Model validation

Model validation was conducted by testing datasets of independent RNA-seq experiments in predicting the biological age. Pearson’s correlation (r), Mean Absolute Error (MAE), and Coefficient of Determination (R^2^) were calculated as measurements indicating performance using “pearsonr”, “np.mean”, and “metrics.r2_score”, respectively (scipy.stats version 1.11.4; numpy version 1.26.2; scikit-learn version 1.3.2).

### Functional enrichment of age-predictive genes and their co-expressed genes

A gene co-expression matrix was constructed from gene expression data of 350 whole blood samples, calculating expression-expression correlations using “pandas.DataFrame.corr” with “pearsonr” option (pandas version 2.1.3). The top five highly co-expressed genes with 36 age-predictive genes were selected for downstream analysis. All 13,834 genes after the preprocessing based on their expression level have been used as the background gene set. ShinyGO (version 0.81) [52] was used to functionally annotate genes.

### Dimension reduction using t-SNE

To visualize the distinct expression patterns across age groups and sex, we employed t-distributed Stochastic Neighbor Embedding (t-SNE), a dimensionality reduction technique that preserves local structure in high-dimensional data, in this case, gene expression data. The analysis was performed on the expression levels of 36 age-predictive genes across 350 blood samples used in training the age prediction model. The pre-processed expression counts were transformed into 2D t-SNE embeddings using the “TSNE.fit_transform” function with “n_components=2” as an option (sklearn.manifold version 1.3.2).

### Transcriptomic age acceleration (TAA)

Transcriptomic Age Acceleration (TAA) is the difference between predicted (transcriptomic) and chronological age at which the blood was drawn from a sample. Prediction error confidence intervals were determined using “sem” (scipy.stats version 1.11.4) and tested for significance using two-tailed, one-sample t-tests using “ttest_1samp” (scipy.stats version 1.11.4).

### Trajectories of TAA across infection stages

The trajectories of transcriptomic age acceleration (TAA) were analyzed in Korean COVID-19, Caucasian COVID-19 ARDS, and Caucasian HCV cohorts across infection stages, as measured by each study. In the Korean COVID-19 cohort, patients were stratified by C-reactive protein (CRP) levels (High and Low). High serum CRP level was defined as CRP > 1mg/dL, and low as CRP ≤ 1mg/dL. In the Caucasian COVID-19 ARDS cohort, patients were classified by mortality: survivor and non-survivor. In the Caucasian HCV cohort, patients were divided according to patient outcome: resolution and chronic disease. Two-sided Welch’s t-tests was performed to obtain nominal P-values distinguishing age acceleration at each stage of infection, using “ttest_ind” (scipy.stats version 1.11.4) with equal_var = False. In addition to the t-tests, a mixed-effects regression model (random intercepts and fixed slopes) was employed to account for individual variability and fixed effects, using “mixedlm” (statsmodels version 0.14.0). We tested the significance of interaction effects between infection stage and disease outcome on TAA. Wald’s test was used to assess the significance of the regression coefficients, with a p-value threshold of < 0.05 considered statistically significant.

### Stage-specific differentially expressed genes (DEGs) in COVID-19

Raw read counts estimated from RSEM were compared between 48 COVID-19 subjects longitudinally collected and 350 healthy bloods in the training data at acute (N=48), mid (N=45), and late (N=41) phases. DESeq2 (version 1.42.0; R package) was used to discover differentially expressed genes using Wald’s test (design: Sample_Trait + Sample_Sex). Those genes with baseMean below 10 were removed. COVID-19 significant gene set (i.e., COVID19) was defined as those genes with statistics of |log2FoldChange|≥1 and FDR < 0.05 while the non-significant gene set (i.e., None) as |log2FoldChange| < 1 and FDR > 0.05. The 36 age predictor genes belong to AgePred gene set. Differences in expression levels were tested using Kruskal-Wallis test with post-hoc Dunn’s test for pairwise comparisons, correcting p-values with “bonferroni” option in “posthoc_dunn” (scikit-posthocs version 0.9.0).

### Clinical correlates of transcriptomic age acceleration (TAA)

Clinical lab values of routine blood tests were correlated with Transcriptomic Age Acceleration (TAA) using “pearsonr” (scipy.stats version 1.11.4). Significance was adjusted for multiple comparisons using “stats.multitest.fdrcorrection” (statsmodels version 0.14.0).

### Calculating TAA in GTEx expression data

We obtained raw GTEx (version 8) gene expression data from the GTEx Portal [53]. We extracted only those samples collected from the whole blood for downstream analysis. Then, DESeq2 normalization was performed using geometric means of genes calculated from the samples of Korean ethnicity, as described previously. TAA was calculated by subtracting the predicted transcriptomic age and the chronological age of the blood donors at the time of enrollment (“AGE”: phv00169063.v9.p2.c1).

### Data availability

Both normalized and un-normalized read count matrices used in the analysis can be found in our GitHub page: https://github.com/korean-genomics-center/transcriptomic_clock. Raw sequencing data and materials used in the study are available from the corresponding author upon request. Public RNA-seq data used in this study, GSE134080 [54], GSE273149 [55], and GSE119117 [56], can be found in Gene Expression Omnibus. Raw expression data from the GTEx project are available at the Portal: https://www.gtexportal.org/home/downloads/adult-gtex/bulk_tissue_expression. Donor information from the GTEx project can be accessed through the dbGaP website (accession number: phs000424.v9.p2).

### Code availability

The codes used to generate data and calculate statistics, as well as the respective readme files, are openly available in the GitHub page: https://github.com/korean-genomics-center/transcriptomic_clock.

## Supplementary Material

Supplementary Figures

Supplementary Table 1

Supplementary Tables 2 and 3

Supplementary Table 4

Supplementary Tables 5 and 6

Supplementary Table 7

Supplementary Table 8

Supplemental Table 9
