# Supplementary material for: Dynamic and reversible transcriptomic age shifts induced by COVID-19 in Korean whole blood
Source: Aging (Albany NY). 2025 Jun 10;17(6):1484–510. doi: 10.18632/aging.206270 (PMC12245201; doi:10.18632/aging.206270)
Supplement: Supplementary Figures [file aging-17-206270-s001.pdf]

SUPPLEMENTARY FIGURES

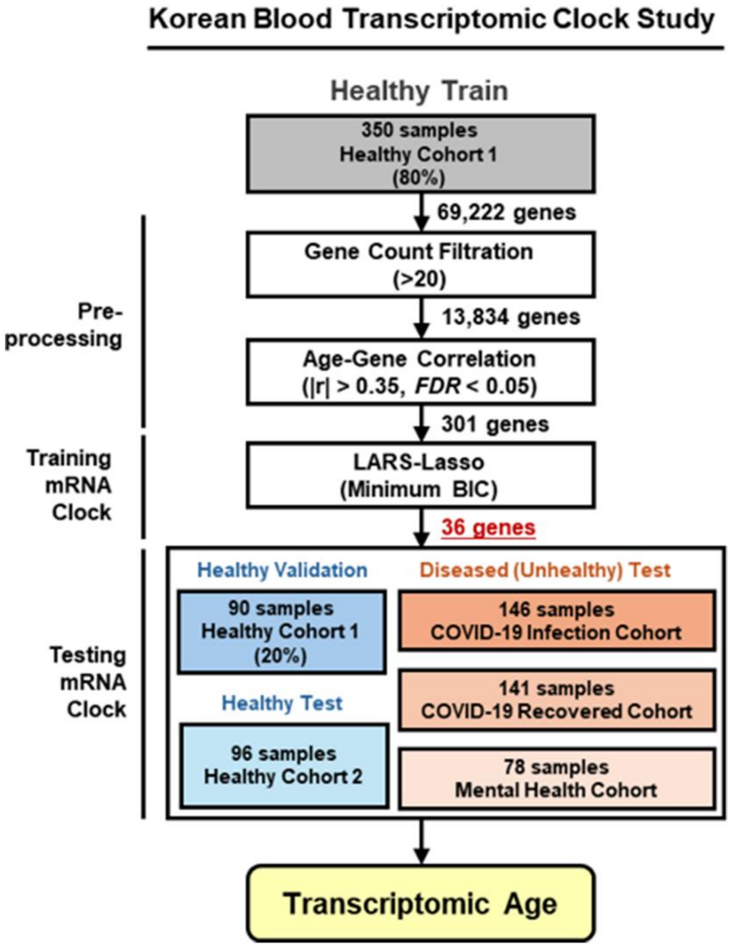

Supplementary Figure 1. Schematic overview of the Korean blood transcriptomic aging clock study. The flow chart illustrating the analytic processes used to derive transcriptomic age from RNA-seq data, which consists of three main steps: pre-processing data, training, and testing the mRNA clock. Boxes in white represent the core algorithm for each step. The number of genes filtered at each stage is indicated following each step. A total of 901 RNA-seq samples were categorized into six cohorts: Healthy Cohort 1 (training set, N = 350; validation set, N = 90), Healthy Cohort 2 (testing set, N = 96), COVID-19 Infection Cohort (N = 146), COVID-19 Recovered Cohort (N = 141), and Mental Health Cohort (N = 78). These cohorts were utilized for training, testing, and evaluating the model, with the COVID-19 and Mental Health cohorts analyzed for biological age changes in response to disease conditions.

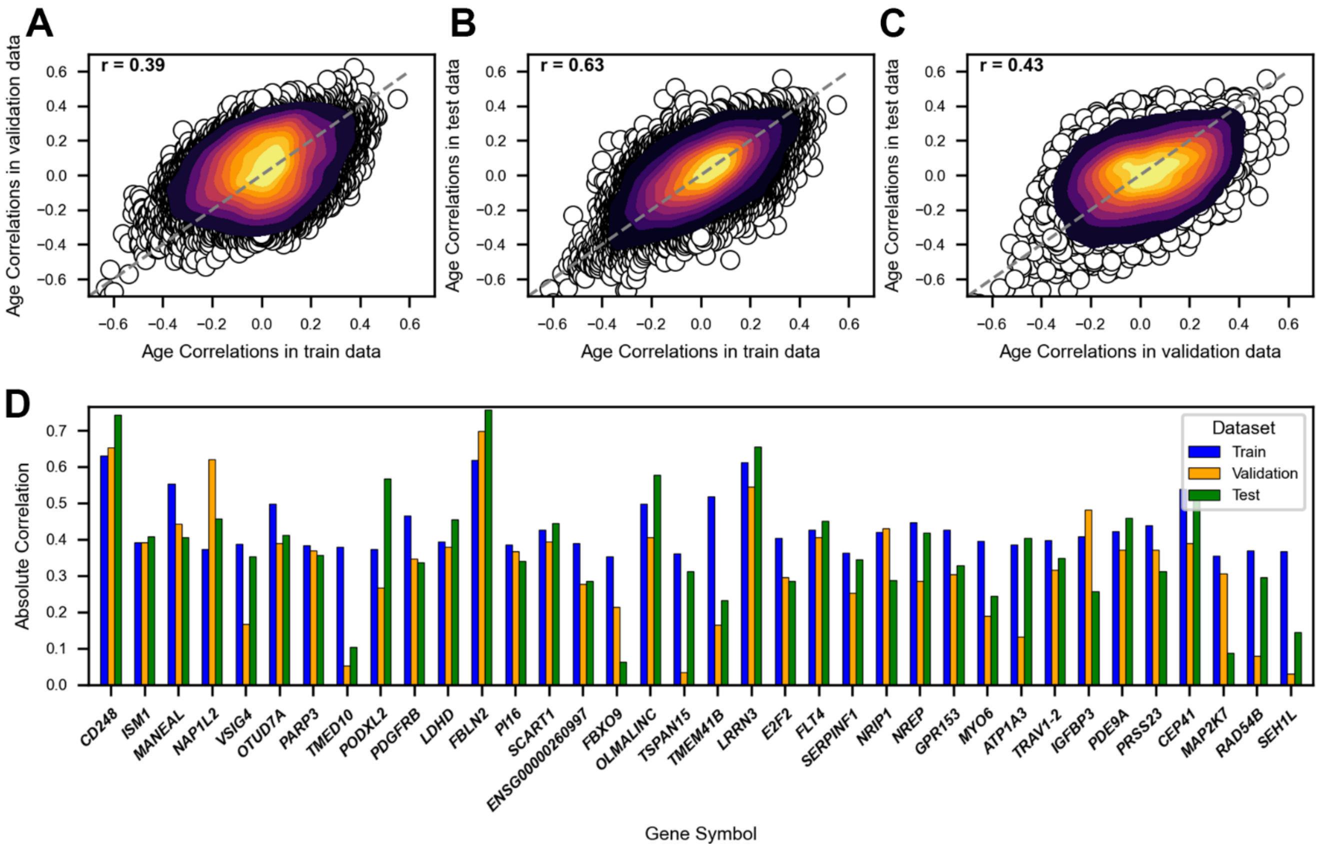

Supplementary Figure 2. Correlation and bar plots showing age correlations of 13,834 stably expressed genes. (**A**–**C**) Scatter plots showing age correlation of 13,834 genes between (**A**) train and validation, (**B**) train and test, (**C**) validation and test data. The x- and y-axes represent Pearson's r of each gene with chronological age (i.e., Age Correlations). (**D**) Bar plots comparing absolute age correlation of 36 age predictors within healthy cohorts. The x-axis lists gene symbols of the predictors, while the y-axis shows absolute value of Pearson's r with chronological age.

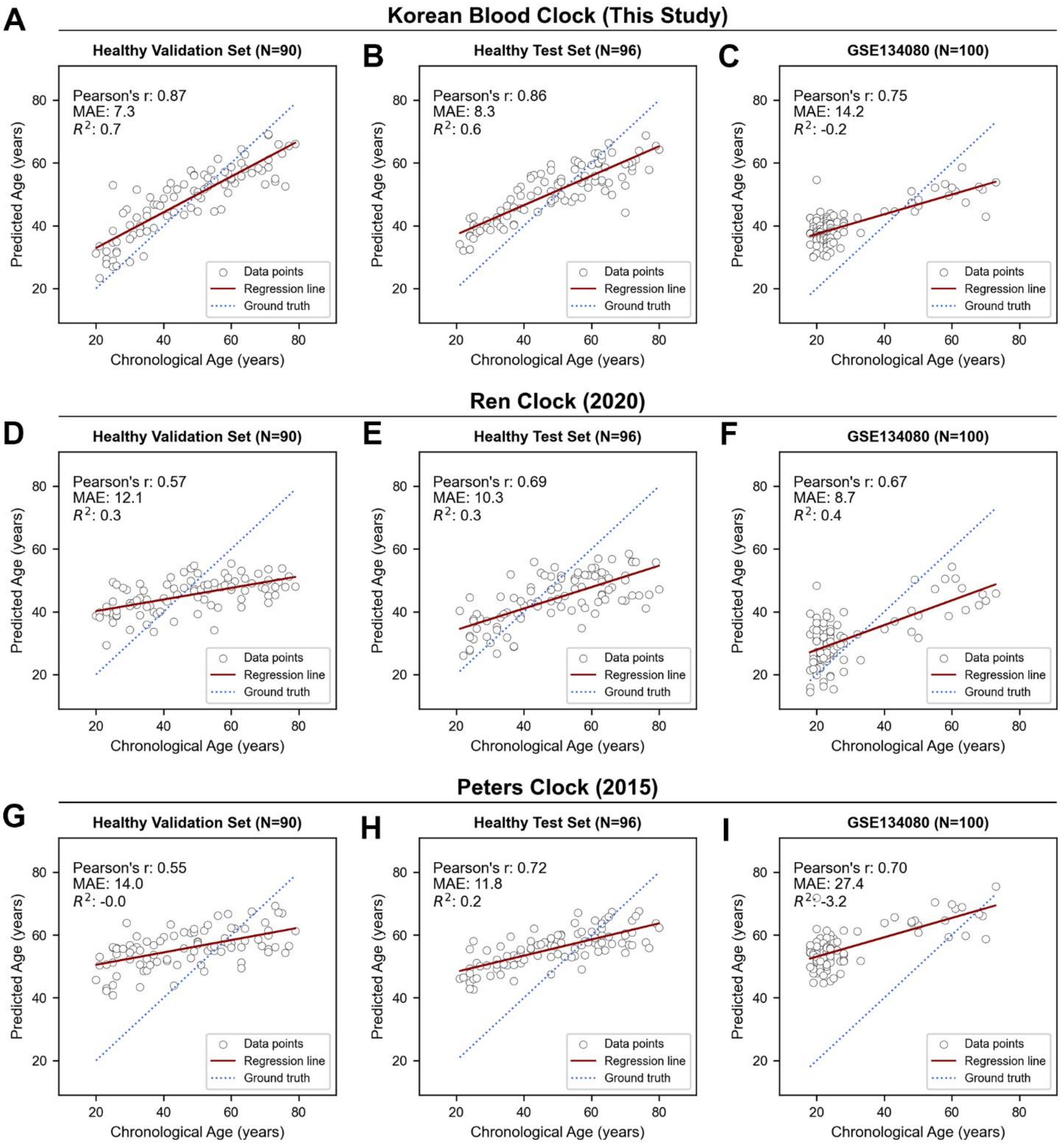

Supplementary Figure 3. Comparing the performance of transcriptomic aging clocks. (**A**–**I**) Scatter plots illustrating the performance of aging clocks: (**A**-**C**) Korean Blood Clock (This Study), (**D**–**F**) Ren Clock (2020), and (**G**–**I**) Peters Clock (2015). The x-axis corresponds to chronological age (in years), and the y-axis displays predicted age via the mRNA clock (in years). Each open grey dot represents a sample. The dotted line in blue shows perfect correlation, while the solid line in red represents a linear regression line indicating the general trend of predicted biological age across chronological age. Pearson's r = Pearson's Correlation; MAE = Mean Absolute Error; R^2 = Coefficient of determination.

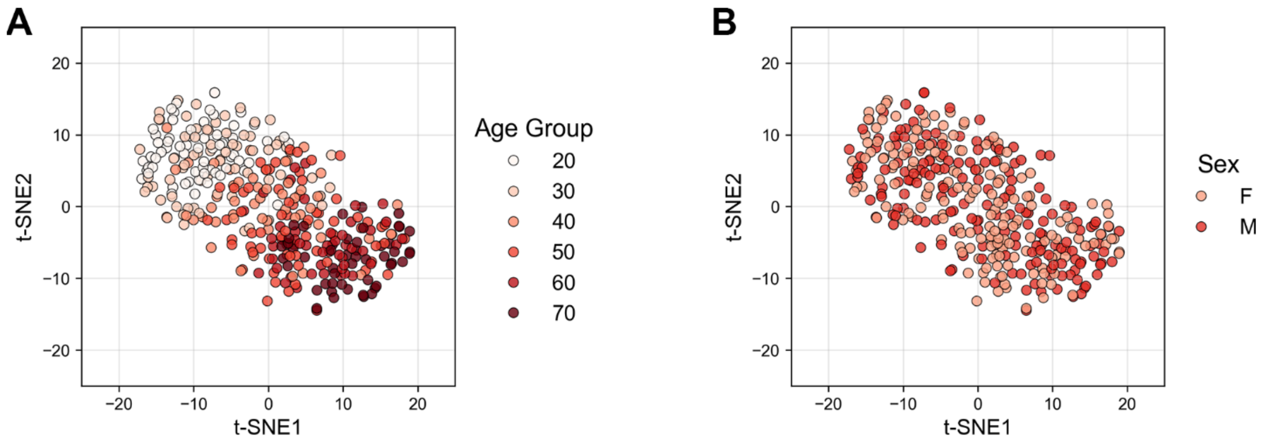

Supplementary Figure 4. Scatterplots of t-SNE results showing the distribution of samples based on transcriptomic data. Each point represents an individual sample, with colors indicating (**A**) age groups (20s to 70s) and (**B**) sex (F: Female, M: Male) of the individual. The axes, t-SNE1 and t-SNE2, are the first and second dimensions of the t-SNE embedding.

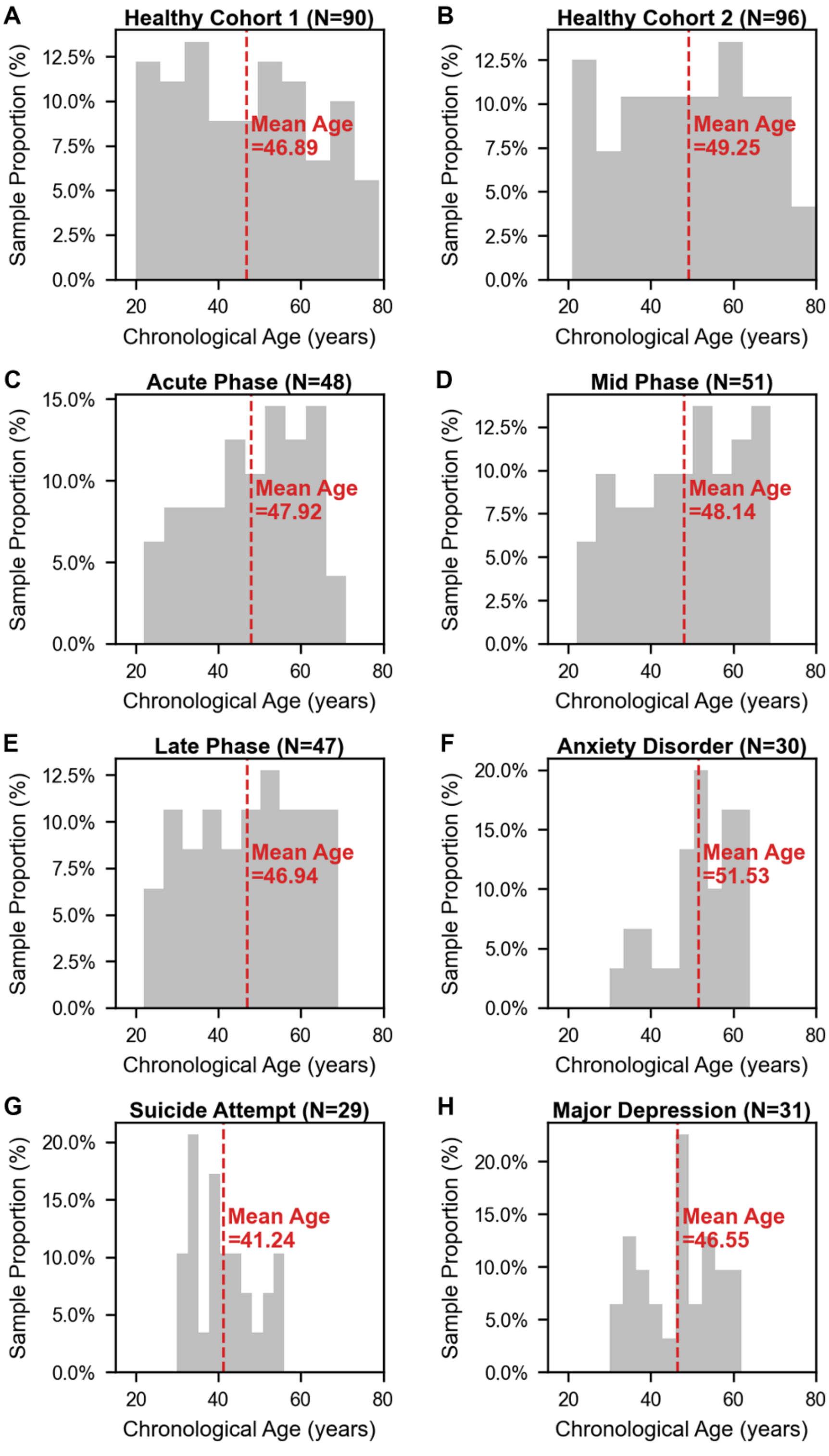

Supplementary Figure 5. Age distributions of study cohorts. Histograms depict the proportional distribution of chronological age across eight distinct study cohorts. (**A**) Healthy Cohort 1 (Validation Set), (**B**) Healthy Cohort 2 (Test Set), (**C**–**E**) COVID-19 patients, and (**F**–**H**) mentally ill patients. The x-axis represents the sample age group in years. The y-axis denotes the sample proportion in percentage. A dashed vertical red line and statistics represent the mean age of the study cohorts overall. The sample size for each cohort is indicated in parentheses.

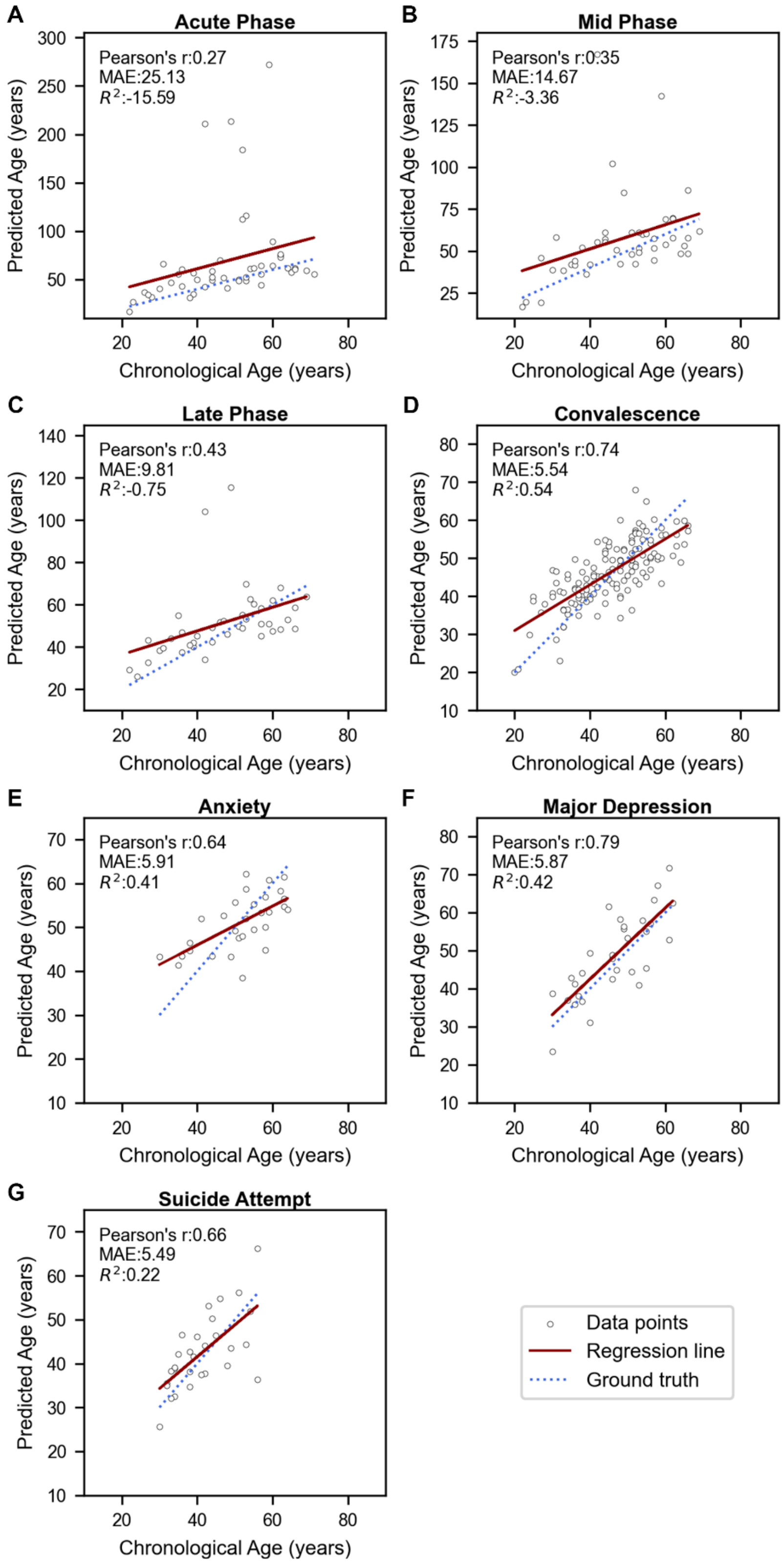

Supplementary Figure 6. Correlation plots showing the variable prediction accuracies across disease phenotypes. Scatter plots illustrate the performance of the age prediction model on (**A**–**D**) COVID-19 patients, and (**E**–**G**) mentally ill patients. The x-axis corresponds to chronological age, and the y-axis displays predicted age via the mRNA clock. Each open grey dot represents a sample. The dotted line shows perfect correlation, while the solid line represents a linear regression line indicating the general trend of predicted biological age across chronological age. Pearson's r = Pearson's Correlation; MAE = Mean Absolute Error; R^2 = Coefficient of determination.

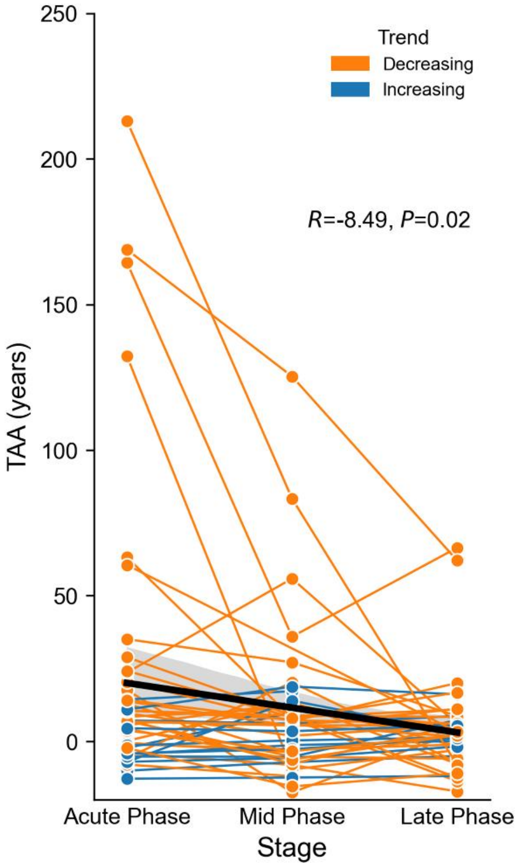

Supplementary Figure 7. TAA dynamics across infection stages of COVID-19. Line plots illustrating the longitudinal changes of transcriptomic age acceleration (in years) for individual COVID-19 patients across infection stages: from acute, mid, to late phase. The green solid line represents the TAA trajectory that is on a decreasing trend, while the red solid line indicates that of an increasing trend. Bold solid line in black shows the overall trend of TAA and 95% CI (Confidence Interval). Numbers of the top right indicate the regression coefficient (*R*) and P-value (*P*).

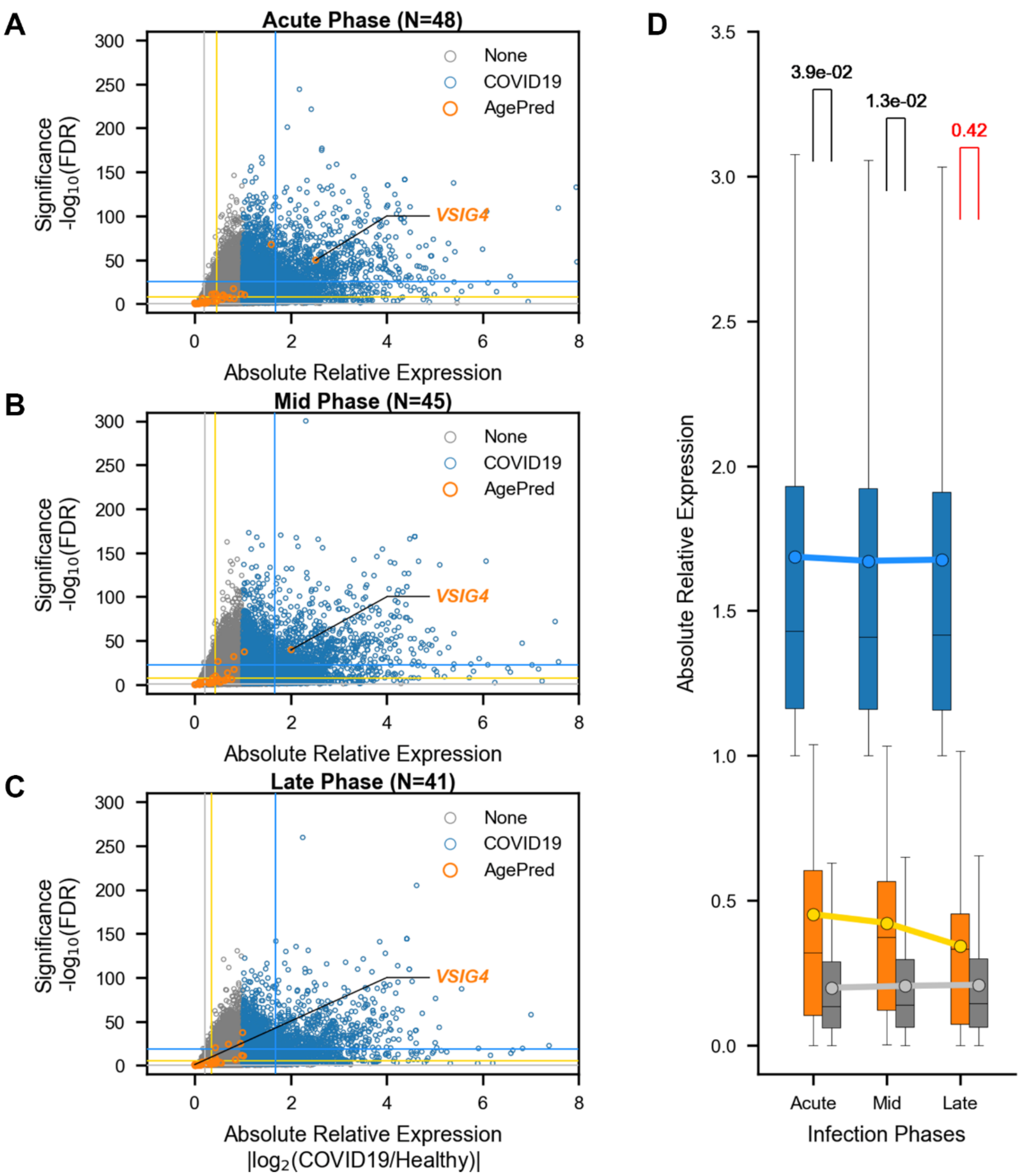

Supplementary Figure 8. Gene expression dynamics of 36 age-predictors in COVID-19 patients. (**A**–**C**) Scatter plots illustrating the relative gene expression in COVID-19 patients at (**A**) acute (N=48), (**B**) mid (N=45), and (**C**) late (N=41) phases, compared to healthy controls. The x-axis denotes absolute relative expression (an absolute log2 value of expression in COVID-19 relative to the healthy), while the y-axis is statistical significance (a negative log10 value of FDR). Each open dot is a gene with blue, orange, and grey colors showing 47 age-predictors (AgePred), COVID-19 significant genes (COVID19; |log2FoldChange| ≥ 1 & FDR < 0.05), and non-significant genes (None; |log2FoldChange| < 1 & FDR >0.05), respectively. Solid lines with corresponding colors represent the mean values of each gene set. (**D**) Bar plots comparing the relative gene expression of each gene set across different infection phases. A filled dot represents the mean relative expression in each phase with solid lines portraying the trend of overall relative expression across time. The statistics represent Bonferroni-corrected p-values of post-hoc Dunn's test between AgePred and None groups. The red figure means no statistical significance.

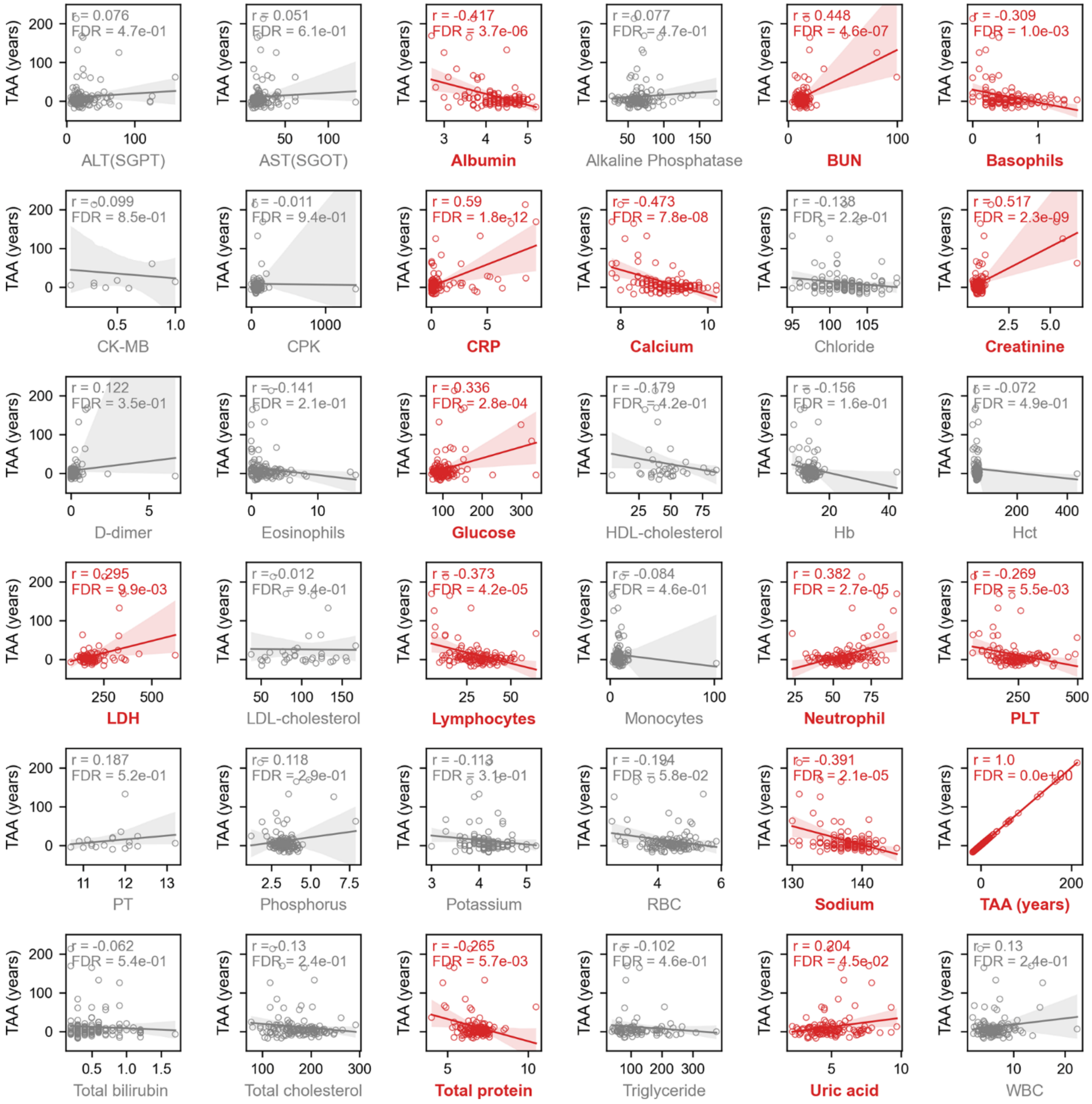

Supplementary Figure 9. Clinical correlates of transcriptomic age acceleration (TAA). Scatter plots depicting the relationship between transcriptomic age acceleration (TAA) and various routine blood biomarkers among COVID-19 patients (N=188) across different stages of infection. Clinical biomarkers are presented in alphabetical order. Significant correlations (*FDR* < 0.05) are highlighted in red, while non-significant associations are shown in gray. Each point represents an individual patient, with Pearson's correlation coefficient (r) and the associated false discovery rate (*FDR*) displayed for each panel. ALT = Alanine aminotransferase; AST = Aspartate transferase; BUN = Blood urea nitrogen; CK-MB = Creatine Kinase-MB; CPK = Creatine phosphokinase; CRP = C-reactive protein; HDL = High-density lipoprotein; Hb = Hemoglobin; Hct = Hematocrit; LDH = Lactate dehydrogenase; LDL = Low-density lipoprotein; PLT = Platelet count; PT = Prothrombin time test; RBC = Red blood cell; WBC = White blood cell.

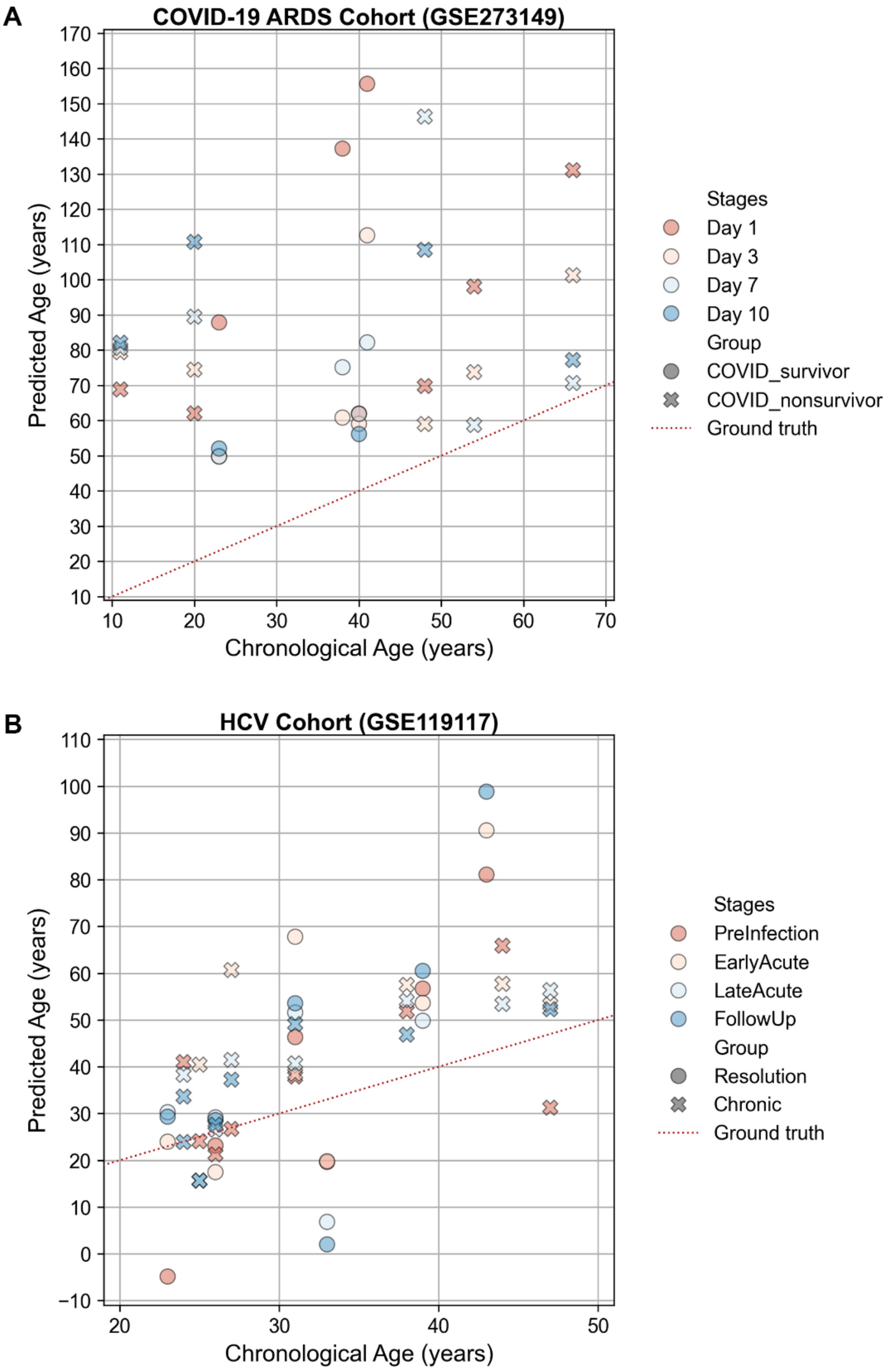

Supplementary Figure 10. Transcriptomic age predictions in COVID-19 ARDS and HCV cohorts across clinical stages. (**A**, **B**) Scatter plots showing predicted transcriptomic age versus chronological age in (**A**) COVID-19 ARDS cohort (GSE273149) and (**B**) HCV cohort (GSE119117). (**A**) Data points are stratified by infection stages (Day 1, Day 3, Day 7, Day 10) and patient outcome groups (COVID survivors and non-survivors). (**B**) Data points are categorized by infection stages (pre-infection, early acute, late acute, follow-up) and clinical outcomes (resolution or chronic infection). The red dashed line in both panels represents the ground truth (chronological age) for reference.

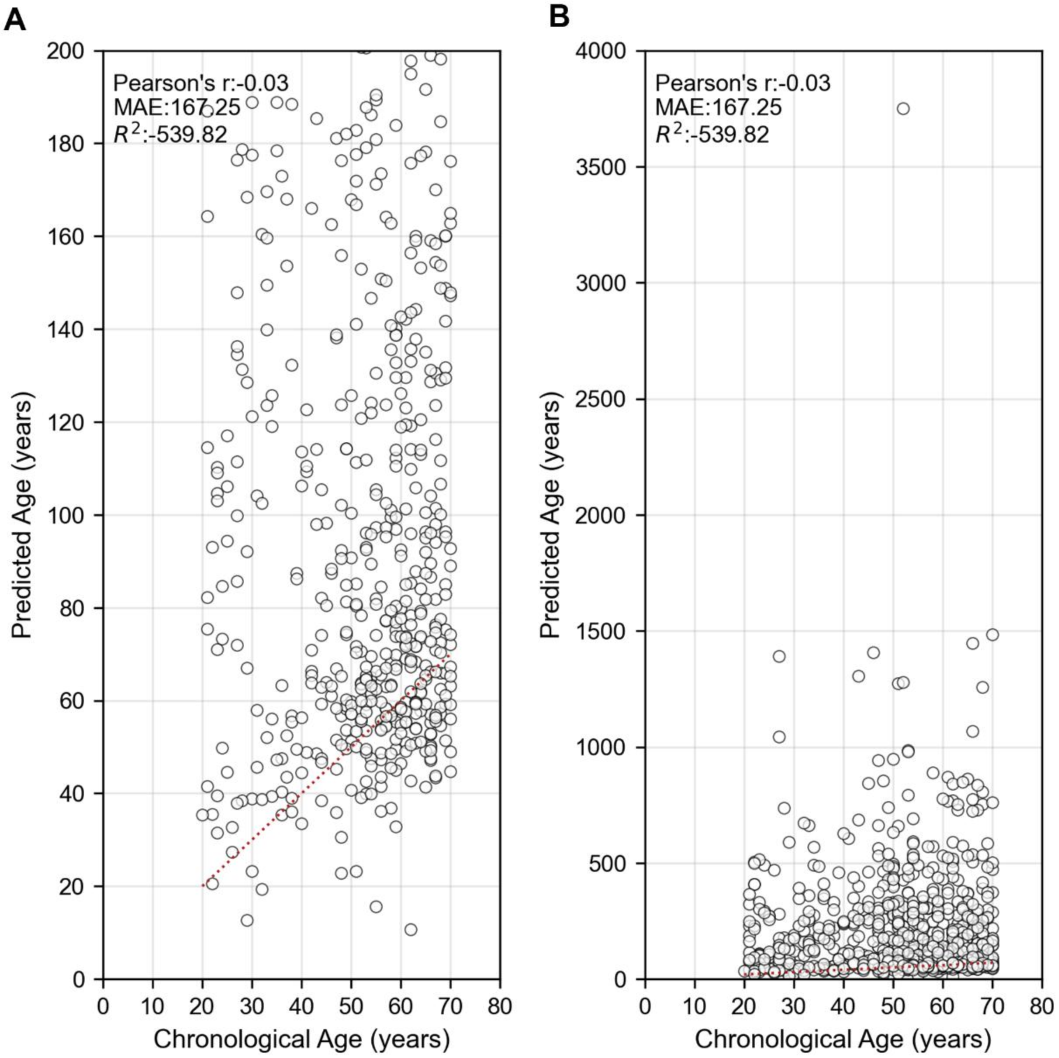

Supplementary Figure 11. Scatterplots illustrating prediction performance in the GTEx dataset. (**A**) Y-axis limited to 200 years, and (**B**) Y-axis extended to 4000 years. Each dot represents a single individual, with predicted transcriptomic age on the y-axis and chronological age on the x-axis. The dotted red line represents the line of perfect correlation. Pearson's correlation coefficient (r), mean absolute error (MAE), and coefficient of determination (R^2) values are shown in each panel.
