# Supplementary material for: Dynamic and reversible transcriptomic age shifts induced by COVID-19 in Korean whole blood
Source: Aging (Albany NY). 2025 Jun 10;17(6):1484–510. doi: 10.18632/aging.206270 (PMC12245201; doi:10.18632/aging.206270)
Supplement: Supplementary Tables 2 and 3 [file aging-17-206270-s003.pdf]

Supplementary Table 2. List of 36 age predictors and their direction and magnitude of effect on aging (negative effect = red ; positive effect = blue ; top 10 = bold).

Index	Selected_Features	GeneSymbol	Regression_Coefficient	Direction
1	ENSG00000174807_CD248	CD248	-2.9794617420466	Negative
2	ENSG00000101230_ISM1	ISM1	1.7593956953689	Positive
3	ENSG00000185090_MANEAL	MANEAL	1.75079853751065	Positive
4	ENSG00000186462_NAP1L2	NAP1L2	1.667382320556	Positive
5	ENSG00000155659_VSIG4	VSIG4	1.63260373130342	Positive
6	ENSG00000169918_OTUD7A	OTUD7A	-1.53095066493412	Negative
7	ENSG00000041880_PARP3	PARP3	1.43266728417743	Positive
8	ENSG00000170348_TMED10	TMED10	-1.40025976163676	Negative
9	ENSG00000114631_PODXL2	PODXL2	-1.31898202194977	Negative
10	ENSG00000113721_PDGFRB	PDGFRB	1.00240951548852	Positive
11	ENSG00000166816_LDHD	LDHD	0.791540152775634	Positive
12	ENSG00000163520_FBLN2	FBLN2	-0.760654210615058	Negative
13	ENSG00000164530_PI16	PI16	0.755391715101398	Positive
14	ENSG00000214279_SCART1	SCART1	-0.709242683601702	Negative
15	ENSG00000260997_ENSG00000260997	ENSG00000260997	0.540382050806283	Positive
16	ENSG00000112146_FBXO9	FBXO9	0.532348714553848	Positive
17	ENSG00000235823_OLMALINC	OLMALINC	-0.525364854338482	Negative
18	ENSG00000099282_TSPAN15	TSPAN15	-0.512990070746724	Negative
19	ENSG00000166471_TMEM41B	TMEM41B	-0.508721506186172	Negative
20	ENSG00000173114_LRRN3	LRRN3	-0.498213516104573	Negative
21	ENSG00000007968_E2F2	E2F2	0.449201563367082	Positive
22	ENSG00000037280_FLT4	FLT4	-0.417806788470162	Negative
23	ENSG00000132386_SERPINF1	SERPINF1	-0.415065379857274	Negative
24	ENSG00000180530_NRIP1	NRIP1	-0.338997686240057	Negative
25	ENSG00000134986_NREP	NREP	-0.300390072179586	Negative
26	ENSG00000158292_GPR153	GPR153	0.298266072957938	Positive
27	ENSG00000196586_MYO6	MYO6	0.230667809902453	Positive
28	ENSG00000105409_ATP1A3	ATP1A3	0.210675590272463	Positive
29	ENSG00000256553_TRAV1-2	TRAV1-2	-0.175410166334397	Negative
30	ENSG00000146674_IGFBP3	IGFBP3	0.174627868762052	Positive
31	ENSG00000160191_PDE9A	PDE9A	-0.154861912233233	Negative
32	ENSG00000150687_PRSS23	PRSS23	0.10418372443051	Positive
33	ENSG00000106477_CEP41	CEP41	-0.0859433567591932	Negative
34	ENSG00000076984_MAP2K7	MAP2K7	0.0638521490271622	Positive
35	ENSG00000197275_RAD54B	RAD54B	-0.0247572566972331	Negative
36	ENSG00000085415_SEH1L	SEH1L	-0.0000827616185103709	Negative

Supplementary Table 3. Summary table of overall means and 95% confidence intervals of transcriptomic age acceleration, and p-values from one-sample t-tests (two-sided) for study cohorts.

Group (Cohorts)	Mean Acceleration [95% CI]	P	FDR
Healthy (Train + Validation + Test)	0.982 [-0.786, 3.584]	0.149702912528978	0.198571192871505
COVID-19 (Acute + Mid + Late Phase)	11.572 [-0.377, 35.040]	0.000084026887234616	0.000252080661703848
Mental Illness (MDD + Anxiety + SA)	0.935 [-3.026, 4.497]	0.198571192871505	0.198571192871505
