# Supplementary material for: Dynamic and reversible transcriptomic age shifts induced by COVID-19 in Korean whole blood
Source: Aging (Albany NY). 2025 Jun 10;17(6):1484–510. doi: 10.18632/aging.206270 (PMC12245201; doi:10.18632/aging.206270)
Supplement: Supplementary Tables 5 and 6 [file aging-17-206270-s005.pdf]

Supplementary Table 5. Summary of mixed-effects model analysis of transcriptomic age association (TAA) across infection stages in COVID-19 ARDS (GSE273149) and HCV (GSE119117) cohorts.

GSE273149	Coef. [95% CI]	Std.Err.	z	P>|z|
Intercept[COVID_nonsurvivor; Day 1]	46.143 [20.494,71.791]	13.086	3.526	0
disease[COVID_survivor]	29.010 [-9.463,67.482]	19.629	1.478	0.139
time[Day 3]	-8.392 [-39.124,22.341]	15.68	-0.535	0.593
time[Day 7]	3.188 [-27.544,33.921]	15.68	0.203	0.839
time[Day 10]	9.300 [-23.753,42.352]	16.864	0.551	0.581
disease[COVID_survivor]*time[Day 3]	-31.677 [-77.776,14.422]	23.52	-1.347	0.178
disease[COVID_survivor]*time[Day 7]	-46.621 [-92.720,-0.522]	23.52	-1.982	0.047
disease[COVID_survivor]*time[Day 10]	-52.610 [-108.389,3.169]	28.459	-1.849	0.065
Group Var	241.568	10.905		
**GSE119117**	**Coef. [95% CI]**	**Std.Err.**	**z**	**P>|z|**
Intercept[Chronic; Pre-infection]	7.093 [-5.375,19.561]	6.361	1.115	0.265
hcvgroup[Resolution]	-8.576 [-27.621,10.469]	9.717	-0.883	0.377
Phase[Early acute]	8.697 [-0.789,18.184]	4.84	1.797	0.072
Phase[Late acute]	2.994 [-5.598,11.586]	4.384	0.683	0.495
Phase[Follow up]	5.588 [-3.004,14.180]	4.384	1.275	0.202
hcvgroup[Resolution]*Phase[Early acute]	4.937 [-8.789,18.664]	7.004	0.705	0.481
hcvgroup[Resolution]*Phase[Late acute]	7.789 [-5.816,21.395]	6.942	1.122	0.262
hcvgroup[Resolution]*Phase[Follow up]	0.926 [-12.199,14.050]	6.696	0.138	0.89
Group Var	246.852	14.681		

The table presents the results of the mixed-effects regression analysis evaluating the association between infection stage, disease outcome (COVID-19 survival and HCV prognosis), and TAAs. The model incorporates random intercepts to account for inter-individual variability. Coefficient estimates (Coef.), standard errors (Std.Err.), z-values, and p-values (P>|z|) are reported for each fixed effect. Wald's test was used to assess the significance of each coefficient, with p-values < 0.05 considered statistically significant. A statistically significant association is highlighted in red.

Supplementary Table 6. CXCL9 is subtly correlated with chronological age in the whole blood of training data.

Cohort name	GeneSymbol	Slope	R^2	Pearsons'r	FDR	Test significant ?
Healthy Train	CXCL9	0.104020911	0.022432609	0.149775195	0.035273179	TRUE
