# Supplementary material for: Dynamic and reversible transcriptomic age shifts induced by COVID-19 in Korean whole blood
Source: Aging (Albany NY). 2025 Jun 10;17(6):1484–510. doi: 10.18632/aging.206270 (PMC12245201; doi:10.18632/aging.206270)
Supplement: Supplemental Table 9 [file aging-17-206270-s008.pdf]

Supplementary Table 9. List of lowest BIC values of the age prediction models across different age-expression correlation coefficient thresholds.

Threshold	alpha	lowest BIC
**0.35**	**1.18598449580599**	**2633.31648787823**
0.36	1.30415998334888	2635.69114442808
0.37	1.28330793080647	2613.7386751641
0.38	1.04188337283776	2634.14497936316
0.39	0.489147006279859	2651.64102744082
0.4	0.848873301009751	2672.56668988947
